# Medulloblastoma in adults: evaluation of the Dutch society for neuro-oncology treatment protocol

**DOI:** 10.1007/s11060-023-04285-8

**Published:** 2023-03-15

**Authors:** L. Bleeker, M. C. M. Kouwenhoven, I. de Heer, B. I. Lissenberg-Witte, A. H. Gijsbers, H. J. Dubbink, J. M. Kros, J. M. M. Gijtenbeek, E. Kurt, C. C. D. van der Rijt, A. T. Swaak-Kragten, F. Y. de Vos, H. L. van der Weide, P. J. French, M. J. van den Bent, P. Wesseling, J. E. C. Bromberg

**Affiliations:** 1 Brain Tumor Center Amsterdam, Department of Neurology, Amsterdam UMC, Amsterdam, The Netherlands; 2 Brain Tumor Center, Department of Neurology, Erasmus MC Cancer Institute, Rotterdam, The Netherlands; 3 Department of Epidemiology and Data Science, Amsterdam UMC, Amsterdam, The Netherlands; 4 The Nationwide Network and Registry of Histopathology and Cytopathology in the Netherlands (PALGA), Houten, The Netherlands; 5 Brain Tumor Center, Department of Pathology, Erasmus MC Cancer Institute, Rotterdam, The Netherlands; 6 Department of Neurology, Radboud University Nijmegen Medical Centre, Nijmegen, The Netherlands; 7 Department of Neurosurgery, Radboud University Nijmegen Medical Centre, Nijmegen, The Netherlands; 8 Department of Medical Oncology, Erasmus MC Cancer Institute, Rotterdam, The Netherlands; 9 Department of Radiation Oncology, Erasmus MC Cancer Institute, Rotterdam, The Netherlands; 10 Cancer Center, Department of Medical Oncology, University Medical Center Utrecht, Utrecht, The Netherlands; 11 University Medical Center Groningen, Department of Radiation Oncology, University of Groningen, Groningen, The Netherlands; 12 Department of Pathology, Amsterdam University Medical Centers/VUmc, Amsterdam, The Netherlands; 13 Laboratory for Childhood Cancer Pathology, Princess Máxima Center for Pediatric Oncology, Utrecht, The Netherlands

**Keywords:** Medulloblastoma, Adults, Treatment, Methylation array, Toxicity

## Abstract

**Purpose:**

Medulloblastoma is a rare tumor in adults. The objective of this nationwide, multicenter study was to evaluate the toxicity and efficacy of the Dutch treatment protocol for adult medulloblastoma patients.

**Methods:**

Adult medulloblastoma patients diagnosed between 2010 and 2018 were identified in the Dutch rare tumors registry or nationwide pathology database. Patients with intention to treat according to the national treatment protocol were included. Risk stratification was performed based on residual disease, histological subtype and extent of disease. All patients received postoperative radiotherapy [craniospinal axis 36 Gy/fossa posterior boost 19.8 Gy (14.4 Gy in case of metastases)]. High-risk patients received additional neoadjuvant (carboplatin-etoposide), concomitant (vincristine) and adjuvant chemotherapy (carboplatin-vincristine-cyclophosphamide) as far as feasible by toxicity. Methylation profiling, and additional next-generation sequencing in case of SHH-activated medulloblastomas, were performed.

**Results:**

Forty-seven medulloblastoma patients were identified, of whom 32 were treated according to the protocol. Clinical information and tumor material was available for 28 and 20 patients, respectively. The histological variants were mainly classic (43%) and desmoplastic medulloblastoma (36%). Sixteen patients (57%) were considered standard-risk and 60% were SHH-activated medulloblastomas. Considerable treatment reductions and delays in treatment occurred due to especially hematological and neurotoxicity. Only one high-risk patient could complete all chemotherapy courses. 5-years progression-free survival (PFS) and overall survival (OS) for standard-risk patients appeared worse than for high-risk patients (PFS 69% vs. 90%, OS 81% vs. 90% respectively), although this wasn’t statistically significant.

**Conclusion:**

Combined chemo-radiotherapy is a toxic regimen for adult medulloblastoma patients that may result in improved survival.

## Introduction

Medulloblastoma is a Central Nervous System (CNS) WHO grade 4 primary brain tumor located in the cerebellum. The incidence of medulloblastoma in adults is approximately 0.6 per 1,000,000 per year, compared to 4.1 per 1,000,000 in children [1, 2]. The 2007 WHO classification of CNS tumors divided medulloblastomas into five histological variants: desmoplastic, classic, anaplastic, large cell and medulloblastoma with extensive nodularity [3]. More recently, various molecular subgroups have been recognized with distinct clinical behavior and outcomes [4, 5]. Methylation-based classification changed the diagnostic approach and made it possible to diagnose brain tumors more precisely [6]. In the 2021 WHO classification of CNS tumors, the medulloblastoma types are designated as WNT-activated, SHH-activated/*TP53*-wildtype, SHH-activated/*TP53*-mutant and non-WNT/non-SHH (Group 3/4) [7].

Most treatment protocols for adult medulloblastoma patients are based on studies in children, due to limited availability of (prospective) studies in adults. However, adults tolerate chemotherapy less well than children [8, 9]. In 2010, in order to standardize treatment, the Dutch Society for Neuro-Oncology (LWNO) developed a treatment protocol for adult medulloblastoma patients based on the available literature at a time when only one prospective study on treatment of adult medulloblastoma had been published [10]. Since 2010, the large majority of adult medulloblastoma patients in the Netherlands have been treated according to this national treatment protocol. In this nation-wide study, we report the toxicity and efficacy of the treatment regime described in this protocol using an intention-to-treat analysis and correlate the clinical features and outcome to molecular characteristics.

## Materials and methods

### Patient and tumor material

Adult patients (18 years or older) diagnosed with a medulloblastoma between January 1st 2010 and October 15th 2018 were included if the treatment was intended to follow the national treatment protocol (see below). To collect all data, treated patients were identified from the prospective Dutch rare tumors registry and from the PALGA-database, a Dutch nationwide network and pathology registry. Pathology laboratories send all excerpts (summary of original pathology report including coding lines and pathologist’s conclusion) to this database, which is then encoded by a Trusted Third Party (ZorgTTP) [11]. All patients provided written informed consent and the study was approved by the Medical Ethics Review Committees of the Erasmus Medical Centre (EMC) and other participating institutions (after co-assessment).

### Molecular analysis

FFPE tissue sections, mounted on glass slides were scored by a neuropathologist (MK) to identify areas with high tumor content. Selected areas were macrodissected from adjacent unstained slides (IdH) and DNA was extracted using the QIAamp DNA FFPE Tissue Kit: Cat. No./ID: 56404 and run on Illumina Infinium Human Methylation EPIC arrays to obtain genome-wide DNA methylation profiles. Data were uploaded to the DKFZ/Heidelberg Brain tumor classifier (version 11b4; www.molecularneuropathology.org [12]) to provide methylation (sub)class and copy number variation profiles. Data of non-WNT/non-SHH tumors was additionally uploaded to the Medulloblastoma classifier group 3/4 (version 1.0) to determine the subtype. We included all tumors with a calibrated Classifier score of ≥ 0.84 [13]. For tumors below the cut-off value, we only included samples if there was a plausible explanation for the lower value, such as a rare histological subtype. In SHH-activated medulloblastomas, next-generation sequencing (NGS) was performed to determine the *TP53*-status and other mutations. By using a NGS panel frequently mutated genes could be assessed [14].

### Treatment protocol

**Fig. 1:**
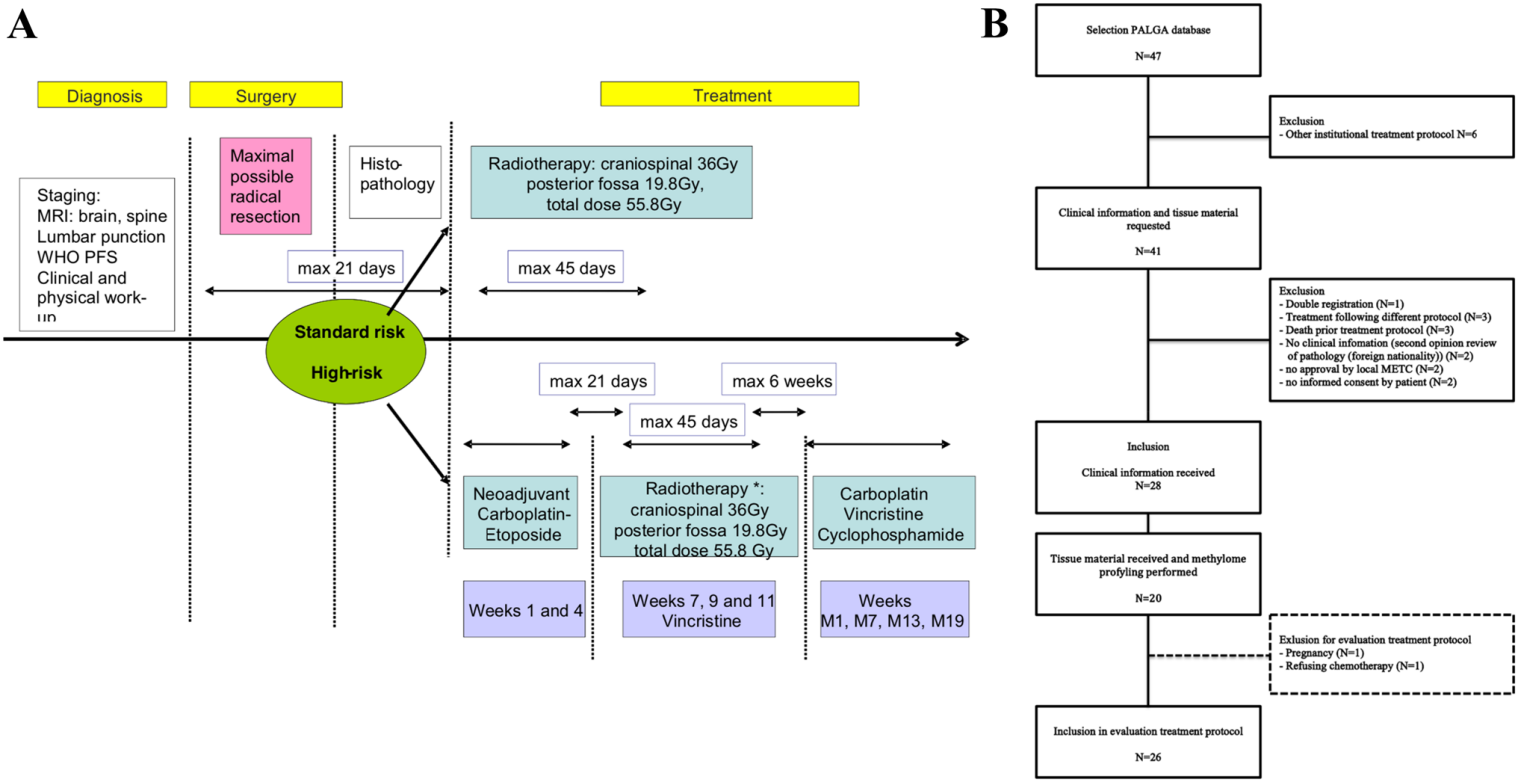
National treatment protocol (**A**) [17] and overview of patient selection process (**B**)

The national treatment protocol is listed in Fig. 1A. In brief, primary treatment, i.e. surgical resection, was followed by a postoperative MRI within 72 h. In case of residual disease of > 1.5 cm^2^, a second look operation within 2 weeks was advised, if judged feasible. We divided patients into risk categories, depending on the presence of residual disease of > 1.5 cm^2^, histological subtype (high-risk in case of large cell/anaplastic phenotype) and extent of disease (presence/absence of metastatic dissemination) [15].

Standard-risk patients received postoperative craniospinal irradiation (CSI), whereas high-risk patients received additional neoadjuvant, concomitant and adjuvant chemotherapy. The radiotherapy protocol consisted of CSI (36 Gy; 20 × 1.8 Gy) and a boost on the posterior fossa (19.8 Gy; 11 × 1.8 Gy), or if present posterior fossa and metastases (14.4 Gy; 8 × 1.8 Gy), with a total dose of 50.4–55.8 Gy. The radiotherapy dosage was equal for standard and high-risk patients. All patients received photon radiotherapy; proton radiotherapy was not available before 2018. Standard-risk patients started radiotherapy as soon as they had recovered from surgery. This should commence within 4 weeks of surgery, but preferably within 21 days. High-risk patients received two courses of neoadjuvant chemotherapy (every 3 weeks) prior to radiotherapy. Chemotherapy consisted of carboplatin IV AUC 6 mg/ml/min on day 1 and etoposide IV 150 mg/m^2^ on days 1–2, preferably starting within 21 days of surgery. Radiotherapy combined with concomitant vincristine (IV 1.5 mg/m^2^ (max. 2 mg) every 2 weeks) was aimed to start within 3 weeks of the second neoadjuvant course. Patients started with adjuvant chemotherapy after recovering from radiotherapy, preferably within 4–6 weeks after the last fraction. Post-radiation chemotherapy consisted of four 42-day courses of chemotherapy, with carboplatin IV AUC 6 mg/ml/min on day 1, vincristine IV 1.5 mg/m^2^ (max. 2 mg) on days 1, 8 and 15, and cyclophosphamide 750 mg/m^2^ on days 22 and 23 (Fig. 1A). Toxicity was evaluated using the Common Terminology Criteria for Adverse Events (CTCAE) version 4.0 [16]. Dose delays and reductions were collected. Toxicity and dose-intensity analysis was limited to patients who received the first course or fraction of the advised therapy.

### Statistical analysis

Patient demographics, tumor and treatment characteristics were summarized using descriptive statistics for quantitative data (median with range, count and percentage). All statistical analyses were performed using SPSS (Version 22). We performed survival analyses for standard/high-risk groups and risk groups per methylation subgroup using Kaplan–Meier analysis. Progression-free (PFS) and overall survival (OS) were defined as the time from date of surgery until date of first event (progression/relapse) or death due to all causes, respectively. A log-rank test was used to test the significant difference. An independent samples t-test was used to test the significant difference between age groups. A *p*-value of < 0.05 was considered significant.

## Results

### Patient characteristics

Twenty-nine adult medulloblastoma patients were registered in the Dutch rare tumors registry between January 1, 2010 and October 15, 2018. Eighteen patients were additionally identified in the PALGA-database. Six patients had to be excluded because they were treated in a hospital that followed another treatment protocol. Another 13 cases were excluded for multiple reasons (see Fig. 1B). Thirty-two patients were treated following the treatment protocol. We received the clinical information and tumor material of 28 and 20 patients, respectively, from 10 neuro-oncology centers. The selection process is shown in Fig. 1B.

Baseline and treatment characteristics are shown in Table 1. Eighteen patients were male (64%). Median age was 29.5 years (range 18–46). At time of diagnosis, twenty-three patients (82%) had a good performance status (Karnofsky performance score 70–100). The histological variants were mainly classic (43%) and desmoplastic (36%). Twelve patients (43%) were classified as high-risk. One patient was 17 weeks pregnant at time of diagnosis.

**Table 1:** Baseline and treatment characteristics

	Number of patients	%	Median	Range
Age (median and range in years)				
18–46	28	100	29.5	18–46
Gender				
Male	18	64	–	–
Female	10	36	–	–
Karnofsky Performance Score				
100	1	4	–	–
90	12	43	–	–
80	7	25	–	–
70	3	11	–	–
60	3	11	–	–
Not reported	2	7	–	–
WHO-ECOG performance status score				
0	2	7	–	–
1	19	68	–	–
2	6	21	–	–
Not reported	1	4	–	–
Localization (several possible)				
Cerebellum	27	96	–	–
Leptomeningeal	4	14	–	–
Brainstem	3	11	–	–
Spinal cord	1	4	–	–
Supratentorial	1	4	–	–
Cerebrospinal fluid	4	14	–	–
Extra-neural metastases				
Present	0	0	–	–
Absent	10	36	–	–
Examination not performed	18	64	–	–
Histological diagnosis				
Classic	12	43	–	–
Desmoplastic/nodular	10	36	–	–
Anaplastic	1	4	–	–
Large cell	0	0	–	–
Medulloblastoma with extensive nodularity	0	0	–	–
Not otherwise specified	5	18	–	–
Molecular subgroups				
WNT-activated	1	5	–	–
SHH–activated	12	60	–	–
Group 3	1	5	–	–
Group 4	4	20	–	–
Methylation result unreliable	2	10	–	–
Extend of resection				
Total gross resection	14	50	–	–
Residual tumor ≤ 1.5 cm^2^	4	14	–	–
Residual tumor > 1.5 cm^2^	7	25	–	–
Unknown (no MRI performed)	3	11	–	–
CSF results				
Negative	19	68	–	–
Malignant cells	4	14	–	–
Unknown (not performed)	5	18	–	–
Risk group				
Standard-risk	16	57	–	–
High-risk	12	43	–	–
Time to start treatment between (median and range in days)				
Second surgery (if necessary)	4	–	37	1–64
Last operation and start neoadjuvant therapy	9	–	27	17–52
Last operation and start radiotherapy (standard-risk)	16	–	40	20–161
Last operation and start radiotherapy (high-risk)	10	–	71	38–94
Start neoadjuvant chemotherapy and start radiotherapy	9	–	43	42–49
Start radiotherapy and start adjuvant chemotherapy	10	–	75	67–98

### Methylation profiling

**Fig. 2:**
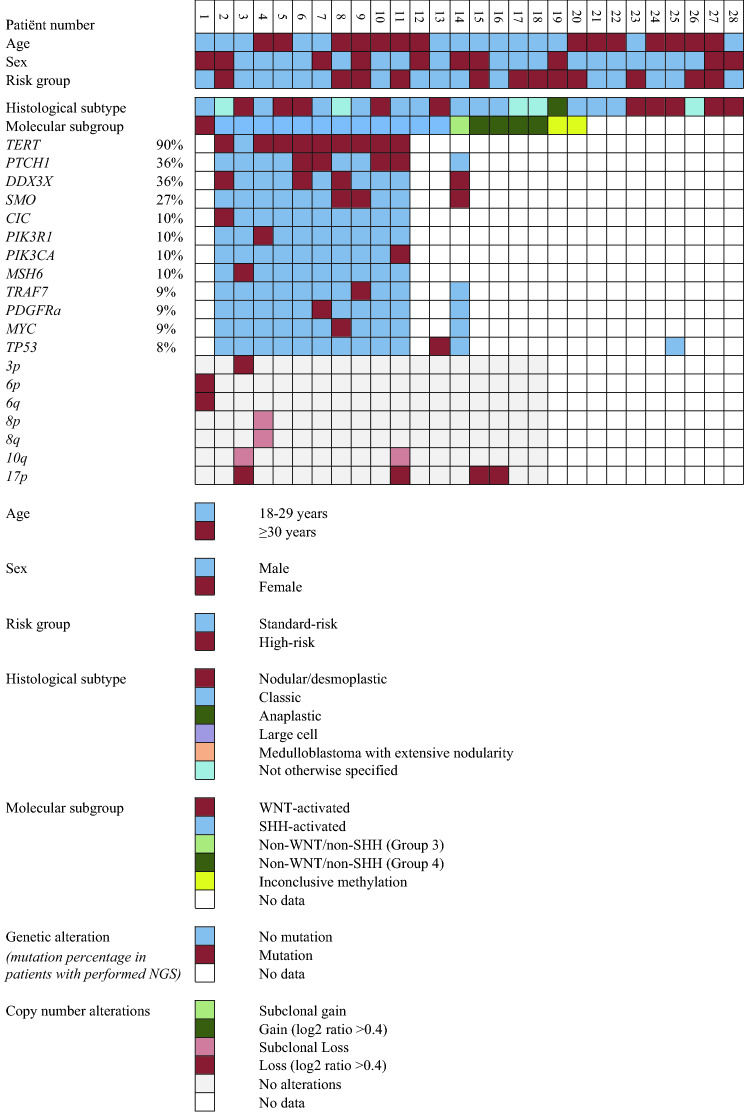
Clinical and histomolecular profiles

Upon methylation profiling the calibrated score for three tumors was below 0.84: one patient had a rare histological subtype, medullomyoblastoma, and was included in our cohort despite the lower Classifier score (0.72). The molecular results of the other two patients were not included. The CNV plot was noisy in one of them suggesting low-quality/technical issues. The most frequent molecular subtype was SHH-activated (60%), followed by non-WNT/non-SHH (25%) and WNT-activated (5%). All desmoplastic medulloblastomas belonged to the SHH-activated subtype. The single WNT-activated tumor had a classic phenotype. The correlation between molecular and histological subtype is shown in Fig. 2. Patients with SHH-activated medulloblastomas were generally older (median 30 years; range 18–44) compared to those with non-SHH-activated tumors (median 23 years, range 18–28), although this difference was not statistically significant (*p* = 0.07).

Two SHH-activated medulloblastomas (17%) had subclonal loss of chromosome 10q and chromosome 17p loss. One of them had additional chromosome 3p loss (8%). No non-WNT/non-SHH tumors had chromosome 8p or 8q losses (Fig. 2). Additional NGS was performed of 10 SHH-activated medulloblastomas. The NGS result of one tumor was unreliable. A *TP53* mutation was found in one of the SHH-activated medulloblastomas. Of additional mutations in SHH-activated tumors, *TERT* promoter mutation was most frequently observed (90%), followed by *PTCH1* (40%), *DDX3X* (30%) and *SMO* mutation (20%; Fig. 2).

### Treatment and toxicities

#### Treatment

Four of the 28 patients underwent a second operation due to residual tumor. Twenty-six of the 28 patients received the advised radiotherapy dose. Two patients received a lower radiation dose than advised in the treatment protocol without reported reason. In one high-risk patient treatment order was changed due to pregnancy (carboplatin/etoposide courses followed by one course carboplatin/vincristine/cyclophosphamide and, after childbirth, radiotherapy), while one high-risk patient refused all chemotherapy. Neoadjuvant chemotherapy was given to 10 of the 12 high-risk patients. One patient with postoperative meningitis was not given neoadjuvant chemotherapy due to long treatment delay. Eight high-risk patients received vincristine during the radiation phase (67%). Besides the already mentioned pregnancy or chemotherapy refusal, in two other patients no reason for omitting vincristine was given. Adjuvant chemotherapy was started according to treatment protocol in 10 (83%) high-risk patients. We excluded the pregnant and chemotherapy refusing patients for postoperative time to treatment and dose delays/reductions evaluation. Table 1 shows the median treatment intervals. In one of four patients with a second operation, this could be performed within the per-protocol specified treatment window. Half of them had a high-risk subtype. Neoadjuvant chemotherapy started within 21 days of surgery in 30% of the patients (median 27, range 17–52). Radiotherapy started within the per-protocol specified treatment window in only 25% of the standard-risk and 30% of the high-risk patients. Median time between surgery and radiotherapy was 40 days (range 20–161) for standard-risk and 71 days (range 38–94) for high-risk patients, with delay mostly brief (median delay for standard-risk 12 days vs. high-risk 8 days). Postoperative infections were the cause of longer delays. In 70% of the patients who received adjuvant chemotherapy, this was started within the per-protocol specified treatment window [median 75 days after start radiotherapy (range 67–98)].

#### Dose delays/reductions

The neoadjuvant chemotherapy dose was reduced in one (11%) and 1-week delayed in two (22%) patients. The dose of the second course was not reported in one patient. Seven of the eight patients receiving vincristine during the chemo-radiation phase received the advised three administrations. Radiotherapy was 1-week delayed in one patient (4%). Only one of 10 patients received the total dose of adjuvant chemotherapy as prescribed in the treatment protocol. The dose was reduced or a part of the chemotherapy was discontinued during the adjuvant chemotherapy due to adverse events in nine patients (90%): treatment reductions were made for two patients in the first course (20%), for five patients in the second course (50%) and for eight patients in the third and fourth courses (80%). Adjuvant chemotherapy was completely discontinued after course three in one patient due to toxicity (10%). Especially vincristine was reduced or stopped early due to neuropathy during adjuvant courses. The delays in adjuvant chemotherapy varied between 0 and 5 weeks (median 1 week).

#### Toxicity

Toxicity could be evaluated in 27 of the 28 patients (Table 2). In one high-risk patient, the radiotherapy phase was not reported. The pregnant patient was only included for the neoadjuvant phase, as the treatment changed afterwards. Hematological toxicity, especially leukopenia (33%) and thrombocytopenia (26%), were most frequently reported CTCAE (v4.0) grade 3–4 toxicity in the whole cohort, thereafter gastro-intestinal toxicity (22%) and infections (15%) were reported. In the high-risk group, hematological toxicity was the only high-grade toxicity reported during the neoadjuvant chemotherapy (leukopenia in 20% and thrombocytopenia in 10%).

**Table 2:** Reported hematological and non-hematological toxicity grades 1/2 and 3/4

Reported adverse events during treatment phase	Grade 1–2	Grade 3–4
Surgery	N	N
Infection	–	3 (11%)
Vertigo	1 (4%)	–

Neoadjuvant chemotherapy	N	N
Leukopenia	–	2 (20%)
Thrombocytopenia	1 (10%)	1 (10%)
Anemia	1 (10%)	–
Ototoxicity	1 (10%)	–

Radiotherapy alone (standard-risk)	N	N
Leukopenia	5 (33%)	1 (7%)
Thrombocytopenia	5 (33%)	–
Infection	1 (7%)	1 (7%)
Gastro-intestinal	3 (20%)	4 (27%)
Fatigue	2 (13%)	–
Alopecia	1 (7%)	–
Cutaneous toxicity	2 (13%)	–

Radiotherapy with concomitant vincristine (high-risk)	N	N
Leukopenia	1 (10%)	1 (10%)
Thrombocytopenia	2 (20%)	3 (30%)
Anemia	1 (20%)	–
Gastro-intestinal	5 (50%)	2 (20%)
Neurotoxicity	4 (40%)	–
Fatigue	1 (10%)	1 (10%)
Alopecia	1 (10%)	–
Cutaneous toxicity	2 (20%)	–
Vertigo	1 (10%)	–
Hiccup	2 (20%)	–

Adjuvant chemotherapy	N	N
Leukopenia	1 (10%)	8 (80%)
Thrombocytopenia	3 (30%)	5 (50%)
Anemia	4 (40%)	2 (20%)
Infection	3 (30%)	–
Neurotoxicity	7 (70%)	–
Nephrotoxicity	–	1 (10%)
Fatigue	1 (10%)	–
Alopecia	2 (20%)	–
Cutaneous toxicity	1 (10%)	–
Vertigo	1 (10%)	–

According to Common Terminology Criteria for Adverse Events (CTCAE) v4.0 [16]

Percentages were calculated as the number of patients with reported adverse event divided by the number of patients receiving specific treatment

Especially high-grade gastro-intestinal toxicity, due to radiotherapy, was reported in the standard-risk group (gastrointestinal toxicity 27%, leukopenia 7% and infection 7%). Whereas in the high-risk group treated with chemoradiation, more high-grade (hematological) toxicity occurred (leukopenia 10%, thrombocytopenia 30%, gastrointestinal toxicity 20% and fatigue 10%). Most adverse events were reported during the adjuvant chemotherapy; in 100% of the patients at least one adverse event was reported at that stage. Grade 3–4 thrombocytopenia and leukopenia were reported in 50% and 80% of the patients, respectively. Eighty percent of the patients had at least one high-grade adverse event during treatment. No fatal adverse events occurred.

### Survival

**Fig. 3:**
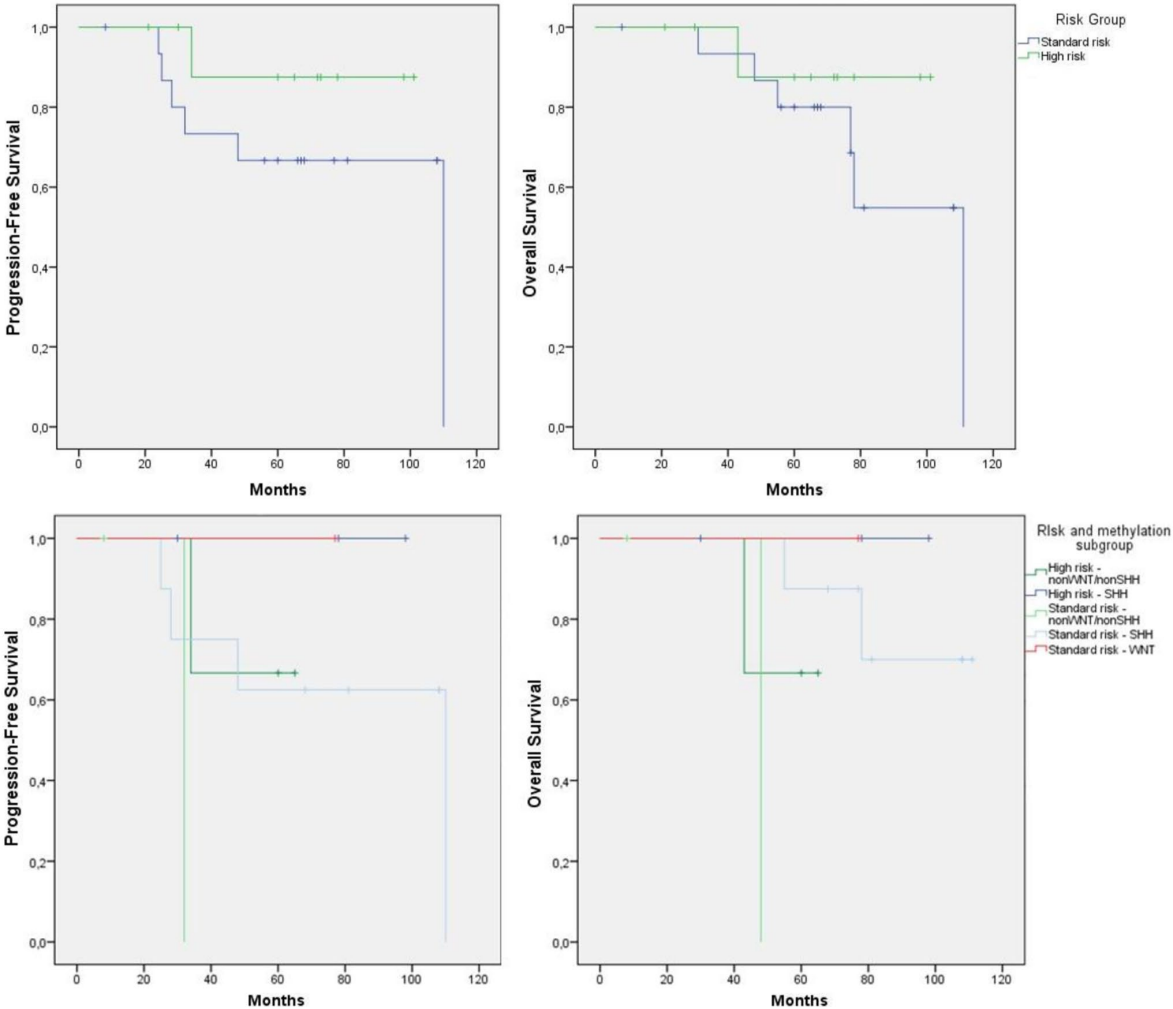
Progression-free survival and overall survival for **A** standard-risk and high-risk groups (log rank PFS p = 0.248 and OS p = 0.358), **B** by risk group per molecular subgroup (log rank PFS p = 0.338 and OS p = 0.120)

The median follow-up time was 67 months (range 8–111). 5-years PFS was 69% for the standard-risk and 90% for the high-risk group (*p* = 0.248). 5-years OS was 81% for the standard-risk and 90% for the high-risk group (*p* = 0.358). Survival for standard-risk patients seemed worse than for high-risk patients, although this difference was not statistically significant. Also, no significant differences were found for the risk group per methylation subgroup (Fig. 3). All patients with either a WNT-activated or high-risk SHH-activated tumor were still alive and without progression at the end of the study period. Three of the five patients with non-WNT/non-SHH tumors had no progression after 5 years (Fig. 3B). Disease progression was seen in one of four patients with a *PTCH1* mutation (25%) and in one of three patients with a *SMO* mutation (33%) after 25 and 32 weeks, respectively. The only patient with a SHH-activated/*TP53*-mutant medulloblastoma showed progression after 4 years.

## Discussion

In this study, we evaluated the toxicity and efficacy of the Dutch national treatment protocol for patients diagnosed with medulloblastoma in adulthood. As in other series [18], the median age of patients was in the late 20 s, the majority of patients had a SHH-activated medulloblastoma and were considered standard-risk. All of our patients received photon-CSI, whereas recent literature showed that proton-CSI leads to less treatment-related morbidity [19]. The chemotherapy treatment schedule was based on previously published regimens for adults but was not tolerated well as only one patient completed all chemotherapy courses. All other patients received adjusted schedules because of toxicity. Especially hematological and gastro-intestinal toxicity were reported frequently. Vincristine was strongly associated with neurotoxicity; early discontinuation during the adjuvant chemotherapy occurred in 50% of the patients. Chemotherapy toxicity also resulted in considerable delays in further treatment. Our data are similar to previous studies showing that adults tolerate chemotherapy poorly compared with children: in the Packer protocol, all adults needed dose reductions due to hematological toxicity, whereas only 14% of the children needed treatment adjustments [8]. More severe (hematological) adverse events were also found in older patients treated in the NOA-07 trial [20], in which all patients were treated with chemotherapy and photon-CSI.

Despite the observed delays in especially radiotherapy in our cohort, survival rates were comparable to published rates. The difference in PFS favoring high-risk patients, a known poor prognostic factor, is intriguing and may be explained by the effects of chemotherapy [18, 21]. The poor tolerance of chemotherapy suggests that our treatment protocol requires adjustments to make it feasible for more patients; the chemotherapy administration may be of greater importance than meeting our per-protocol specified deadlines. Moreover, the difference in survival between the standard-risk and high-risk patients was not significant and PFS and OS were similar to those found by Brandes et al., on which our treatment schedule was based [10]. Studies with (intention to) chemo-radiotherapy for all patients observed 57–68% PFS and 70–89% OS during a follow-up period of 3–5 years [20, 22, 23], also comparable with our findings.

We compared survival between methylation subclasses and found no significant differences. Although this may have been the result of the small number of patients, other series also have failed to identify differences in survival between these categories in adults, as opposed to children[24, 25]. The majority of patients in our cohort had a SHH-subtype (60%). Further molecular stratification of these tumors revealed a high prevalence of *TERT* promoter, *DDX3X*, *PTCH1* and *SMO* mutations and low prevalence of *TP53* mutations, which is in line with previous literature [25, 26]. Previous studies showed that *TP53* mutations are associated with unfavorable outcome [25–27], though the only patient in our cohort showed progression only after 4 years. *TERT* promoter mutations are reported to be associated with a favorable outcome in patients of SHH-activated tumors but a poor outcome in patients with Group 4 tumors [28]. Conflicting data exist regarding prognostic value of *PTCH1* and *SMO* mutations [22, 25]. The majority of patients with *PTCH1* or *SMO* mutations in our cohort survived without progression.

Beside mutations, previous studies in adult patients with SHH-activated tumors have shown that chromosome 3p, 10q or 17p losses were correlated with decreased survival. In contrast, patients with chromosome 8 loss in Group 4 tumors had improved outcomes [22, 25]. In our cohort, the patients with subclonal chromosome 10q loss, chromosome 3p and/or 17p losses survived during the follow-up period. There was no chromosome 8 loss in Group 4 medulloblastomas in our series. Of note, the treatment regimen of our study wasn’t based on molecular stratification.

The strength of our study is the nationwide multicenter consecutive series of patients treated in a uniform manner. Limitations are the relatively low patient numbers and lack of a uniform and standardized registration of adverse events. No significant results were found regarding prognostication of medulloblastoma subgroups. WNT-activated and non-WNT/non-SHH-activated medulloblastomas are underrepresented in adults; this makes prognostication research in these subgroups challenging. However, we showed that toxicity of neoadjuvant chemotherapy led to delay in radiotherapy initiation in more than 2/3 of patients but that nevertheless survival was comparable to that in other series. Moreover, the similar survival shows that chemotherapy is an important modality to consider in medulloblastoma patients [9, 21]. Moots et al. found disease progression in two adult medulloblastoma patients during neo-adjuvant chemotherapy [29]. The Dutch treatment protocol was amended recently for standard-risk patients to radiotherapy combined with concomitant and adjuvant chemotherapy [30]. Also, proton-CSI is now used, which leads to less bone marrow depression and therefore allows for more dose-intense adjuvant chemotherapy administration [19]. Some of the frequent mutations, such as those activating the SHH pathway, hold promise for more tailored treatment. SMO inhibitors, as selective antagonist of this pathway, will be investigated in a prospective trial [31]. Further molecular characterization and discovering new targetable mutations can be appealing steps towards more personalized treatment. For the development of robust risk stratification, international collaboration is required because of the rarity of the medulloblastoma in adults.

## Data Availability

Raw data that supports the finding of this study are available from the corresponding author, upon reasonable request.
